# Prevalence of adverse events following T-cell redirecting therapies in patients with metastatic castration-resistant prostate cancer: a pooled analysis

**DOI:** 10.1093/oncolo/oyag172

**Published:** 2026-05-02

**Authors:** Abhiraj Saxena, Marc Yorker, Nicholas A Zorko, Vivek Narayan, Bilal A Siddiqui, Biren Saraiya, William J Tester, Patrick J Mille, Amy L Shaver, Nikita Nikita, Grace L Lu-Yao, William Kevin Kelly, Kevin K Zarrabi

**Affiliations:** Department of Medical Oncology, Sidney Kimmel Cancer Center, Thomas Jefferson University, Philadelphia, PA 19107, United States; Division of Medical Oncology, Rutgers Robert Wood Johnson Medical School, New Brunswick, NJ 08901, United States; Department of Medical Oncology, Sidney Kimmel Cancer Center, Thomas Jefferson University, Philadelphia, PA 19107, United States; Department of Medicine, Masonic Cancer Center, University of Minnesota-Twin Cities, Minneapolis, MN 55455, United States; Department of Medical Oncology, Abramson Cancer Center and Perelman School of Medicine, University of Pennsylvania, Philadelphia, PA 19104, United States; Department of Genitourinary Medical Oncology, Division of Cancer Medicine, The University of Texas MD Anderson Cancer Center, Houston, TX 77030, United States; Division of Medical Oncology, Rutgers Robert Wood Johnson Medical School, New Brunswick, NJ 08901, United States; Department of Medical Oncology, Sidney Kimmel Cancer Center, Thomas Jefferson University, Philadelphia, PA 19107, United States; Department of Medical Oncology, Sidney Kimmel Cancer Center, Thomas Jefferson University, Philadelphia, PA 19107, United States; Department of Medical Oncology, Sidney Kimmel Cancer Center, Thomas Jefferson University, Philadelphia, PA 19107, United States; Department of Medical Oncology, Sidney Kimmel Cancer Center, Thomas Jefferson University, Philadelphia, PA 19107, United States; Department of Medical Oncology, Sidney Kimmel Cancer Center, Thomas Jefferson University, Philadelphia, PA 19107, United States; Department of Medical Oncology, Sidney Kimmel Cancer Center, Thomas Jefferson University, Philadelphia, PA 19107, United States; Department of Medical Oncology, Sidney Kimmel Cancer Center, Thomas Jefferson University, Philadelphia, PA 19107, United States

**Keywords:** T-cell engagers, bispecific antibodies, chimeric antigen receptor t-cell therapy, safety, prostate cancer

## Abstract

**Background:**

T-cell redirecting therapies, including T-cell engagers (TCEs) and Chimeric Antigen Receptor T-cell (CAR-T), are under investigation in metastatic castration-resistant prostate cancer (mCRPC). Despite their promising therapeutic potential, these therapies remain in early stages of development due to treatment-related adverse events (TRAEs). This study aims to quantify the frequency and severity of adverse events in clinical trials with a pooled analysis.

**Materials and Methods:**

A systematic search of PubMed, CINHAL, Scopus, and Ovid was conducted to identify clinical trials evaluating TCEs or CAR-T in patients with mCRPC. Types and frequencies of TRAEs were collected and analyzed using a random-effects model.

**Results:**

Thirteen TCEs trials involving 861 patients and 5 CAR-T trials involving 55 patients were identified. Cytokine release syndrome occurred in 59% (95% confidence interval: 25, 86) and 43% (28, 59) with TCEs and CAR-T, respectively. Neurologic TRAEs occurred in 39% (22, 60) with CAR-T compared to 9% (4, 17) with TCEs (*P* < 0.0001). Hematologic and hepatic TRAEs occurred in 34% (8, 75) and 25% (12, 44) with CAR-T, compared to 23% (7, 56) and 33% (12, 64) with TCEs, respectively. Musculoskeletal/dermatologic TRAEs occurred in 33% (24, 42) and 29% (8, 58) with TCEs and CAR-T, respectively. Renal and gastrointestinal TRAEs occurred in 21% (5, 51) and 14% (2, 43) with CAR-T, compared to 15% (8, 25) and 21% (12, 36) with TCEs, respectively.

**Conclusions:**

These data underscore the TRAE profiles associated with these therapies and emphasize the importance of differentiating off-target toxicities from immune effector cell—toxicities to inform the development of effective TRAE mitigation strategies.

Implications for Practice While early-phase clinical trials of T-cell therapies in metastatic castration-resistant prostate cancer have shown encouraging outcomes, their use can in some cases be limited by treatment-related adverse events, leading to increased use of biologics and steroids, longer hospital stays, and a higher risk of mortality. We found that patients treated with CAR-T therapies experienced significantly higher rates of all-grade neurological treatment-related adverse events (including immune effector-cell-associated neurotoxicity syndrome), grade ≥3 cytokine release syndrome, and grade ≥3 hepatic TRAEs compared with those receiving T-cell engagers. Overall, however, the rates and severity of toxicities observed to date are consistent with those reported for FDA-approved therapies in hematologic malignancies. Future studies should compare CAR-T-cells and T-cell engagers by target antigen to determine whether specific antigens are associated with an increased risk of off-target toxicity.

## Introduction

Prostate cancer (PC) is the most commonly diagnosed malignancy among men, with over 310 000 new cases diagnosed annually in the United States, accounting for more than 35 000 deaths each year.^1^ Global epidemiologic data indicate comparable incidence and mortality, with approximately 1.2 to 1.6 million new cases and over 350 000 annual deaths reported worldwide.^2^^,^^3^ The incidence of metastatic PC continues to rise. Given the incurable nature of advanced disease, drug development continues to evaluate novel classes of safe and effective therapies aimed at prolonging life.^4^^,^^5^

Androgen ablation—using nonsteroidal anti-androgens, luteinizing hormone-releasing hormone agonists and antagonists, and androgen pathway inhibitors—remains the cornerstone of treatment for patients with locally advanced or metastatic PC. However, progression into a castrate-resistant state is an almost universal outcome, after which life-prolonging therapies are employed in a primarily palliative setting.^6–8^ These therapies include androgen receptor signaling inhibitors, taxane- or platinum-based chemotherapy regimens, radioligand therapies, and poly(ADP-ribose) polymerase inhibitors. Despite their therapeutic benefits, these agents often exhibit limited tissue selectivity, significant off-target effects, high toxicity, and limited durability of response.^9^

Research has focused on evaluating the therapeutic potential of immune-oncology (IO) approaches in treating metastatic PC. However, clinical trials investigating various IO strategies, including studies evaluating immune checkpoint inhibitors, have largely failed to demonstrate a meaningful survival benefit for most patients.^10^^,^^11^ Limited outcomes are frequently attributed to the unique characteristics of the PC tumor microenvironment (TME), which is described as “cold” or an immune desert. The TME is characterized by a low tumor mutational burden and an abundance of immunosuppressive cell populations, including tumor-associated macrophages and regulatory T-cells, which collectively reduce the availability of neoantigens necessary for immune recognition and a subsequent response.^12^ The immunosuppressive milieu contributes to T-cell anergy, enabling tumor immune evasion and therapeutic resistance. Addressing these challenges has become a key focus in advancing PC therapies, and recent progress has highlighted the potential of strategies to enhance T-cell activation.

T-cell redirecting strategies, including bispecific T-cell engagers (TCEs) and chimeric antigen receptor (CAR) T-cells, are currently under investigation in patients with metastatic PC. T-cell engagers offer several advantages over conventional monoclonal antibody-based approaches. These laboratory-engineered constructs contain two distinct antigen-binding fragments specific to a single antigen.^13^ The dual specificity enables TCEs to simultaneously target both a tumor-associated antigen and a receptor on CD4+ or CD8+ T-cells. Consequently, CD3+ TCEs can act as a molecular “bridge,” facilitating the recruitment of cytotoxic immune cells into the typically immunologically “cold” TME, enabling MHC-independent tumor cell killing.^14^ CD28+ TCEs similarly enhance T-cell activation but through co-stimulatory signaling after TCR-MHC engagement.^15^ Chimeric antigen receptor T-cell therapy represents another promising immunotherapeutic approach, involving *ex vivo* modification of a patient’s autologous T-cells to express synthetic receptors targeting tumor antigens.^16^ Several early-phase clinical trials are currently underway to evaluate the safety and efficacy of CAR-T therapy in metastatic castration resistant PC (mCRPC).^17^

Despite their promising therapeutic potential, T-cell-engaging therapies remain in the early stages of development. Preliminary trial results have highlighted both challenges and successes regarding safety and efficacy. One major obstacle in the clinical administration of these therapies is their associated safety profile, which includes fatalities, particularly cytokine release syndrome (CRS), immune effector-cell-associated neurotoxicity syndrome (ICANS), and on-target-off-tumor (OTOT) toxicity.^18^ Due to small sample sizes of TCEs and CAR-T therapy trials in mCRPC, an understanding of the incidence and severity of these toxicities as a class of therapy remains incomplete in this disease population. This systematic review and meta-analysis aims to combine and quantify adverse events (AEs) from a series of clinical trials in a pooled analysis, providing greater clarity on their frequency and severity. By better characterizing the most common and severe adverse reactions, this work may help inform strategies to prevent and manage these toxicities as further trials develop.

## Methods

### Literature search and data extraction

**Figure 1.**
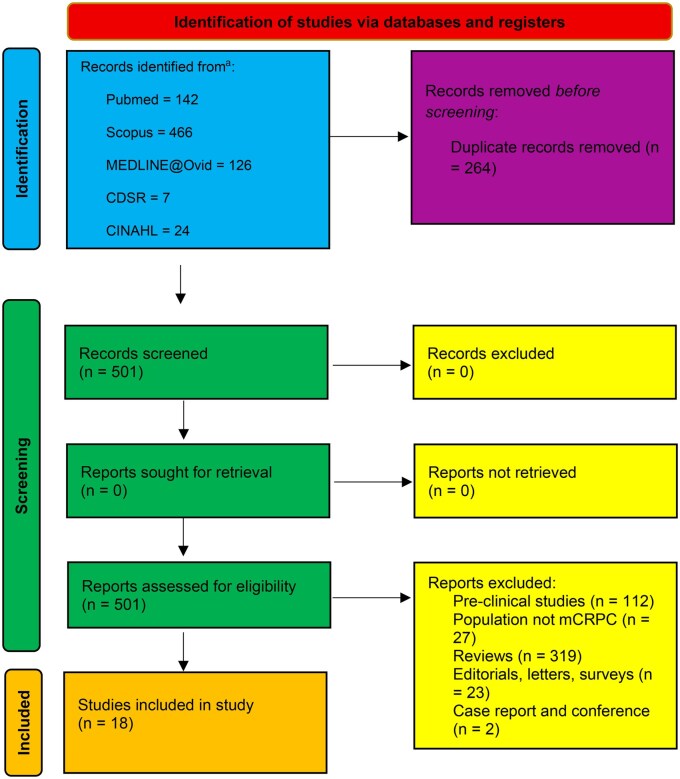
PRISMA diagram. This work is licensed under CC BY 4.0. To view a copy of this license, visit https://creativecommons.org/licenses/by/4.0/. *Source*: Page et al.

A systematic search was conducted in 5 databases: PubMed, Ovid Medline, Cochrane Central Register of Controlled Trials, Scopus, and Cumulative Index to Nursing and Allied Health Literature to identify ongoing or completed phase I or II clinical trials from 1/1/2020 to 9/1/2025 that evaluated TCEs or CAR-T therapy in patients with mCRPC. Our study did not require institutional review board approval and followed the PRISMA statement guidelines for meta-analysis and systematic reviews (Figure 1). Exclusion criteria included patients younger than 18, nonhuman subjects, editorials, and commentaries. MESH search terms were as follows: (“PC” OR “Prostate tumor” OR “Prostate neoplasm”) AND (“bispecific antibodies” OR “bispecific monoclonal antibody” OR “TCEs” OR “bispecific antibody” OR “bispecific monoclonal antibody” OR “CAR-T” OR “Chimeric Antigen Receptor T-cell”).

Relevant demographics, oncological and treatment history, rates of treatment-related adverse events (TRAEs), response rates, and outcomes were extracted into an Excel spreadsheet. Treatment-related AEs were grouped by relevant system to pool data.

### Statistical analysis

Statistical analyses were done using R software, version 3.5.1 (R Foundation for Statistical Computing, Vienna, Austria). Clinical data were reported as pooled means with 95% confidence intervals (CIs), with forest plots. Continuous data were pooled using a random-effects model, while categorical variables were analyzed using meta-analysis of proportions with logistic transformations. Statistical significance was determined with *P*-values less than or equal to 0.05, and study heterogeneity was evaluated with I^2^.

## Results

### Description of studies and patient characteristics

Thirteen trials evaluating TCEs in 861 patients and 5 trials evaluating CAR-T therapies in 55 patients were selected (PRISMA, Figure 1).^19–36^ The characteristics of the trials are summarized in Table 1. The average age in the TCEs cohort was 67 years (95% CI: 63, 71), and in the CAR-T therapy cohort was 66 years (60, 72). Six trials in the TCEs cohort studied prostate-specific membrane antigen (PSMA) x CD3 constructs, 1 trial studied a STEAP1 x CD3 TCE, 1 studied a PSMA x CD28 TCE, 1 studied a PSMA x Vy2 TCE, 1 studied a HER2 x CD3 TCE, 1 studied a DLL3 x CD3 TCE, 1 studied a TMEFF2 x CD3 TCE, and 1 studied a KLK2 x CD3 TCE. Four trials in the CAR-T cohort studied PSMA CAR-T, and 1 studied PSCA CAR-T.

**Table 1:** study characteristics.

Study name	Author, year	Immunotherapy	Treatment arm/cohort 1	Control arm/cohort 2	Number of patients	Endpoints	Median follow-up, month	Outcomes
**Role for IL2 adjunctive co-therapy for suppression of a solid tumor with designer T cells: Phase I trial data in prostate cancer**	Junghans^19^	PSMA CAR-T	Chemotherapy + 10^9^–10^10^ autologous T-cells escalation dosing IV with low dose IL2 infusion	None	5	Overall response rate (ORR) Prostate-specific antigen (PSA) decline Plasma IL2 Engraftment rate	N/A	40% (2/5) had a partial response 20% engraftment target exceeded in 60% (3/5) of patients
**Pasotuxizumab, a BiTE® immune therapy for castration-resistant prostate cancer: Phase I, dose–escalation study findings**	Hummel et al.^20^	PSMA × CD3	Pasotuxizumab SC (starting dose 0.5 μg doses of 0.5-172 μg)	Pasotuxizumab cIV (starting dose of 5 μg 5-80 μg/d)	47	Safety Decline in PSA ORR Biomarkers	N/A	68% (21/31) in subQ had a decline in PSA; 88% (14/16) in cIV cohort 17% (5/30) stable in subQ; 19% (3/16) in ciV 23% (7/30) in SC had partial response; 82% (13/16) ciV had partial response 26% (8/31) SC had grade >3 TRAE, 44% (7/16) cIV had stage >3 TRAE 0% SC CRS, 19% (3/16) in cIV
**Results of an ongoing phase 1/2a dose escalation study of HPN424, a tri-specific half-life extended prostate-specific membrane antigen (PSMA)-targeting T-cell engager, in patients with metastatic castration-resistant prostate cancer (mCRPC)**	De Bono et al. ^21^	PSMA × CD3	Fifteen cohorts with target dose of HPN424 from 1.3-160 ng/kg fixed dose; up to 300 ng/kg with step dosing after priming dose	None	80	Safety Circulating tumor cell (CTC) levels Decline in PSA	24 weeks	4% (3/80) with grade 3 CRS; 63% (50/80) with all-grade CRS 57% (32/56) with a reduction in CTC 21% (13/63) with a decline in PSA
**Safety and early efficacy results from a phase 1, multicenter trial of PSMA-targeted armored chimeric antigen receptor T-cells (CAR T-cells) in patients with advanced mCRPC**	McKean et al.^22^	PSMA CAR-T	Cohort 1 (*n* = 2): CAR-T 1-3 × 10^7^ cells Cohort 2 (*n* = 4): CAR-T 1-3 × 10^8^ cells	Cohort 3 (*n* = 3): 0.7-1 × 10^8^ cells + anakinra prophylaxis	9	Safety Cytokine levels PSA decline ORR	N/A	22% (2/9) with grade 5 multiorgan failure 4/7 had declines in PSA 2/5 PSA50 rate 4/5 with stable disease
**Phase 1 study of P-PSMA-101 CAR-T-cells in patients with mCRPC**	Slovin et al.^23^	PSMA CAR-T	3 day fludarabine + cyclophosphamide then dose escalation of 0.25-15 × 10^6^ cells/kg	None	14	Safety PSA decline PSMA-positron emission tomography (PET)	N/A	71% (10/14) had declines in PSA 36% (5/14) PSA50 rate 57% (8/14) all-grade Cytokine Release Syndrome (CRS) and 14% (2/14) all-grade immune effector cell-associated neurotoxicity syndrome (ICANS) rate
**PSMA-targeting TGFβ-insensitive armored CAR T-cells in metastatic castration-resistant prostate cancer: a phase 1 trial**	Narayan et al.^24^	PSMA CAR-T	Cohort 1: CAR-T-cell infusion 1-3 × 10^7^ Cohort 2: CAR-T-cell infusion 1-3 × 10^8^	Cohort 3: Chemodepletion + CAR-T-cell infusion 1-3 × 10^8^ Cohort -3: Chemodepletion + CAR-T-cell infusion 1-3 × 10^7^	13	Engraftment rate PSA decline ORR Overall survival (OS) Progression-free survival (PFS)	Every 3 months for 2 years, long-term until 15 years	76% (10/13) had declines in PSA 8% (1/13) PSA90 rate, 31% (4/13) PSA30 rate 38% (5/13) with stable disease OS: 56% (6 months), 56% (12 months), 37% (18 months), 37% (24 months), 37% (30 mo); median 15.9 months PFS: 39% (6 months), 8% (12 months); median 4.4 months
**JNJ-70218902 (JNJ-902), a TMEFF2 × CD3 bispecific antibody, in prostate cancer: Initial results from a phase I dose escalation study**	Calvo et al.^25^	TMEFF2 × CD3	9 cohorts receiving increasing doses of JNJ-902	None	73	Safety PSA decline ORR	N/A	5% (4/73) with all grade CRS 11% (8/73) PSA50 rate 5/73 (7%) with partial response
**Safety and preliminary clinical activity of JNJ-63898081 (JNJ-081), a PSMA and CD3 bispecific antibody, for the treatment of mCRPC**	Lim et al.^26^	PSMA × CD3	IV cohorts receiving JNJ-081 from 0.1 μg/kg-3 μg/kg IV JNJ-081	SC cohorts receiving JNJ-081 with no priming ranging from 3 μg/kg-30 μg/kg; priming cohorts with priming dose of 10 μg/kg followed by either 30 μg/kg or 55 μg/kg	39	Safety Decline in PSA Systemic cytokine concentrations ORR	N/A	66.7% (26/39) had all-grade CRS, 0% with grade >3 CRS 13% (2/23) with stable disease 0% decline in PSA in the IV group, 41% (11/27) with a decline in PSA in the SC group Antidrug antibodies in 16.7% (2/12) in the IV group and 63% (17/27) in the SC group
**Early dose escalation of LAVA-1207, a novel bispecific gamma-delta T-cell engager (Gammabody), in patients with mCRPC**	Mehra et al.^27^	PSMA × Vy2	LAVA-1207 1.5 µg starting dose IV, every 2 weeks with target dose of 40 µg	None	16	Safety ORR	N/A	No treatment discontinuation for any treatment-emergent adverse event No CRS noted 3/8 with stable disease
**Phase II trial of pembrolizumab and anti-CD3 × anti-HER2 bispecific antibody armed activated T-cells in mCRPC**	Vaishampayan et al.^28^	HER2 c CD3	Cohort received 8 infusions of HER2 BATs (up to 10^10^/infusion) twice a week for 4 weeks with pembrolizumab every 3 weeks	None	14	Safety ORR PFS OS Change in immune and cell markers	36 months	14% (2/14) with grade ≥ 3 fatigue; 7% (1/14) with grade ≥ 3 mental status change 71% (10/14) with declines in PSA 38% (5/13) with stable disease 1/13 with progressive disease Median PFS: 5 months Median OS: 31.6 months
**Xaluritamig, a STEAP1 × CD3 XmAb 2 + 1 immune therapy for mCRPC: Results from dose exploration in a first-in-human study**	Kelly et al.^29^	STEAP1 c CD3	7 Low dose cohorts ranging from 0.001 to 0.3 mg weekly IV JNJ-081	7 High dose cohorts with step-wise dosing 0.1 to 2 mg IV JNJ-081	97	Safety Decline in PSA ORR	Median: 8.1 months	60% (27/45) in low dose cohorts with all-grade CRS; 83% (43/52) in high dose cohorts 40% (17/43) PSA50 rate in low dose cohorts; 59% (26/44) PSA50 in high dose cohorts 8% (19/43) PSA90 rate in low dose cohorts; 36% (16/44) in high dose cohorts 60% (18/43) in low dose cohorts with stable disease; 38% (14/44) in high dose cohorts with stable disease
**A phase I study of Acapatamab, a half-life extended, PSMA-targeting bispecific T-cell engager for mCRPC**	Dorff et al.^30^	PSMA × CD3	Short-term IV target dose of acapatamab every 2 weeks in a 28-day cycle, with increasing target dose with step dosing to prevent CRS	Extended IV infusion over 2-5 days on D1, then target dose on D8, then target dose every 2 weeks	133	Safety Tolerability Decline in PSA ORR Radiographic progression-free survival (rPFS)	Median of 5.6 months	97.7% (130/133) with all-grade CRS 9.8% (13/133) with PSA90 rate; 31.6% (42/133) with PSA50 rate 37.9% (25/66) with stable disease; 10.6% (7/66) with partial response; 37.9% (25/66) with progressive disease rPFS: 3.8 mo
**Phase 1 clinical trial of AMG 340, a PSMA-targeted T-cell engager with a novel low-affinity CD3 binding domain designed to mitigate toxicity for the treatment of mCRPC**	Falchook et al.^31^	PSMA × CD3	AMG 340 (1.5-800 mg IV every 3 weeks during dose escalation stage	None	42	Safety Decline in PSA ORR	6 months	2% (1/41) with grade >3 CRS; 52% (22/41) with all-grade CRS 14% (6/42) with grade >3 thrombocytopenia; 25% (11/42) with all-grade thrombocytopenia 10% (4/41) PSA50 rate 52% (14/27) with stable disease
**Updated results on the bispecific PSMAxCD3 antibody CC-1 for treatment of prostate cancer**	Heitmann et al.^32^	PSMA × CD3	Dose escalation to target 826 µg and dose expansion	None	28	Safety Decline in PSA		13% (19/28) with all-grade CRS 25% (15/61) PSA50 rate 16% (10/61) PSA90 rate
**Prostate stem cell antigen (PSCA)-CAR T-cell therapy in metastatic castration-resistant prostate cancer: a phase 1 trial**	Dorff et al.^33^	PSCA CAR-T	Cohort 1 (*n* = 3): CAR-T without chemodepletion Cohort 2 (*n* = 6): CAR-T with chemodepletion	Cohort 3 (*n* = 5): CAR-T with reduced chemodepletion	14	CAR-T-cell percentage of total CD3+ cells Safety PSA decline ORR OS	12 months	36% (5/14) with all grade CRS 7/10 had stable disease 7/14 had declines in PSA OS in 6 mo: 33% (cohort 1), 67% (cohort 2), 40% (cohort 3)
**Pasritamig, a first-in-class, bispecific T-cell engager targeting human kallikrein 2, in mCRPC: A phase I study**	Stein et al.^34^	KLK2 × CD3	SC cohorts dose of pasritamig escalating from 0.5-2000 mg every week to every 6 weeks	IV cohorts dose of pasritamig escalating from 150-900 mg every week to every 6 weeks	174	Safety Tolerability rPFS ORR Decline in PSA RP2D efficacy	156 weeks	30.4% (31/102) with all-grade CRS in the SC cohorts; 16.7% (12/72) in the IV cohorts; 8.9% (4/45) in RP2D safety population rPFS: 4.07 mo in SC cohorts; 6.01 mo in IV cohorts; 7.85 mo in RP2D group ORR: 7% (4/57) in SC cohorts; 11.1% (3/27) in IV cohorts; 0/7 in RP2D group 26.5% (27/102) PSA50 rate in SC cohorts; 25% (18/72) in IV cohorts; 36.4% (12/33) in RP2D group
**Updated safety and efficacy results from a phase 1/2 study of nezastomig, a first-in-class co-stimulatory PSMA×CD28 bispecific antibody (bsAb), plus cemiplimab (anti-PD-1) in patients (pts) with mCRPC**	Siddiqui et al.^35^	PSMA × CD28	Monotherapy nezastomig for 3 weeks with doses 0.1-300 mg, then combination with cemiplimab 350 mg IV every 3 weeks	None	78	Safety Tolerability Decline in PSA rPFS OS	Median of 22.7 months	13% (10/78) with all-grade CRS, no CRS event ≥ grade 3 2% (2/78) with grade 5 immune-mediated adverse reactions rPFS: 5 months in DL ≥ 30 mg; 2.1 months in DL < 30 mg OS: 17.3 months in dose-loading (DL) ≥ 30 mg; 10.3 months in DL < 30 mg 25% (15/61) with PSA50 rate; 16% (10/61) with PSA90 rate
**Safety and efficacy of Tarlatamab in patients with neuroendocrine prostate cancer: Results from the phase 1 b DeLLpro-300 study**	Aggarwal et al.^36^	DLL3 × CD3	>1 dose of Tarlatamab 1 mg every 2 weeks until the target dose of 100 mg	None	40	Safety ORR PFS DLL3 expression	N/A	82.5% (33/40) with all-grade CRS; 5% (2/40) with grade 3 CRS 12.5% (5/40) with ICANS and neurological treatment-related adverse event (TRAE); 1/40 with grade 4 TRAE 56.3% (18/32) DLL3+ rPFS: 3.5 months in DLL3+ patients; 1.7 months in DLL3-/unknown patients OS: 9.8 months with DLL3+; 5.5 months with DLL3-/unknown patients 33.3% (6/18) with stable disease in DLL3+ patients; 15.4% (2/13) in DLL3- patients 27.8% (5/18) with progressive disease in DLL3+ patients; 46.2% (6/13) in DLL3- patients

Table 2 summarizes patients’ baseline demographic and oncologic characteristics across TCEs and CAR-T studies. The pooled mean age was 67 years (95% CI: 63,70), with no statistically significant difference between the bispecific and CAR-T subgroups. Most patients self-identified as White, with 84% (55, 96) in the TCEs group and 91% (25, 100) in the CAR-T group. Representation of Black, Asian, and unknown racial backgrounds was limited and comparable between groups. Baseline prostate-specific antigen (PSA) values showed wide variability, with a pooled mean of 397 µg/mL (106 688) among TCEs recipients and a pooled mean of 16 µg/mL (5, 27) in the CAR-T cohort; this difference did reach statistical significance (*P* = 0.0103). Performance status data were available only for the TCEs group, with 53% having ECOG 0 and 47% having ECOG 1.

**Table 2:** Demographics and oncological history.

	T-cell engager therapy				CAR-T therapy				Overall				Subgroup
Variable, mean	Number of studies	Number of patients (*N* or *n*/*N*)	Pooled value (95% CI)	*I* ^2^ (%)	Number of studies	No. of patients (*N* or *n*/*N*)	Pooled value (95% CI)	*I* ^2^ (%)	Number of studies	Number of patients (*N* or *n*/*N*)	Pooled value (95% CI)	*I* ^2^ (%)	*P*-value
**Age, years**	10	700	67 [63, 71]	0	2	27	66 [60, 72]	0	12	727	67 [63, 70]	0	.76
**Race, White (%)**	5	289/465	84 [55, 96]	50	1	12/14	91 [25, 100]	0	6	301/479	86 [62, 96]	25	.73
**Race, Black (%)**	4	18/418	4 [3, 7]	0	1	2/14	9 [0, 75]	0	5	20/432	5 [3, 7]	22	.66
**Race, Asian (%)**	4	58/418	8 [1, 36]	83^a^	1	0/14	0 [0, 100]	0	5	58/432	5 [1, 26]	69	1.00
**Race, unknown (%)**	5	100/465	1 [0, 20]	86^a^	1	0/14	0 [0, 100]	0	6	100/479	1 [0, 15]	79	1.00
**Baseline PSA, ng/dL**	6	492	397 [106, 688]	0	2	27	16 [5, 27]	0	8	519	217 [6, 428]	8	.0103^a^
**ECOG Performance status (%)**													
** 0**	7	279/538	53 [45, 61]	50^a^					7	279/538	53 [45, 61]	50	NA
**1**	7	258/538	47 [39, 55]	50^a^			–		7	258/538	47 [39, 55]	50^a^	NA
**Gleason score (%)**													
** 7 or less (%)**	3	40/100	40 [31, 50]	0	1	6/13	46 [19, 75]		4	46/113	41 [32, 50]	0	.67
** 8 or above (%)**	3	53/100	53 [43, 63]	0	1	5/13	38 [14, 68]		4	58/113	51 [42, 60]	0	.33
** Unknown (%)**	3	7/100	7 [3, 14]	0	1	2/13	15 [2, 45]		4	9/113	8 [4, 15]	0	.31
**Prior hormone treatment (%)**	7	499/544	98 [88, 100]	90^a^	2	24/27	99 [11, 100]	0	9	523/571	98 [88, 100]	85	.89
**Prior chemotherapy (%)**	9	611/666	98 [91, 100]	38	2	18/27	73 [40, 92]	0	11	629/693	97 [88, 100]	42	.009^a^
**Prior taxane chemotherapy (%)**	7	464/544	88 [69, 96]	78^a^	2	18/27	73 [40, 92[	0	9	482/571	86 [70, 94]	73	.30
**Prior radiotherapy (%)**	5	87/326	16 [4, 51]	83^a^	1	9/13	69 [39, 91]	–	6	96/339	21 [5, 56]	83	.02^a^
**Other therapies (%)^b^**	3	33/100	22 [2, 77]	83^a^	1	10/13	77 [46, 95[	–	4	43/113	31 [5, 79]	80	.08
**Metastatic sites (%)**													
** Bone**	4	232/319	73 [51, 88]	92^a^	2	15/27	56 [37, 73]	0	6	247/346	67 [49, 81]	86	0.22
** Lymph Nodes**	4	134/319	45 [28, 64]	83^a^	2	18/27	67 [47, 82]	0	6	152/346	53 [37, 68]	75	0.12
** Visceral^c^**	6	157/478	38 [20, 60]	92^a^	2	6/27	22 [10, 41]	0	8	175/505	33 [20, 50]	86	0.23
** Unclassified**	4	119/438	4 [0, 44]	92^a^	–	–	–	–	4	119/438	4 [0, 44]	92	NA

a Significant data heterogeneity present (*P* ≤ .05).

b Includes atezolizumab, dexamethasone, dutasteride, granulocyte macrophage colony stimulating factor, niraparib, olaparib, pembrolizumab, sunitinib, sipuleucel, zibotentan, estramustine, prednisone, and zoledronic acid.

c Includes liver, peritoneum, and bladder.

Regarding the Gleason score, the majority had high-grade disease: 53% of the TCEs group and 38% of the CAR-T group had a score ≥8. The proportion of unknown Gleason scores was low and similar between cohorts. Prior treatment history indicated near-universal exposure to androgen ablation, androgen receptor signaling inhibitors, and chemotherapy, particularly in the TCEs group (98% and 98%, respectively), whereas only 73% of the CAR-T group had received chemotherapy (*P* = 0.009). Prior taxane use was common in both groups (88% vs 73%; *P* = 0.30). Radiotherapy exposure differed significantly between groups, with 16% of the TCEs patients receiving radiation compared to 69% in the CAR-T group (*P* = 0.02). Use of other therapies—including agents such as pembrolizumab, olaparib, and zoledronic acid—was more frequent in the CAR-T group (77% vs 22%), though not statistically significant.

Sites of metastases were broadly similar between groups. Bone was the most common site (67% overall), followed by lymph nodes (53%) and visceral organs (33%), with no significant difference in metastatic site distribution observed between treatment modalities. Notably, several variables demonstrated substantial heterogeneity, particularly ECOG status, prior taxane and radiation exposure, and metastatic involvement of bone and lymph nodes, as reflected by high I^2^ values and *P*-values >0.05.

### Treatment-related adverse events

#### Cytokine release syndrome

Table 3 summarizes the pooled toxicity profiles for each treatment subgroup, grouped by organ system. Cytokine release syndrome was observed in 59% (95 CI: 25, 86) of patients treated with TCE compared to 43% (28, 59) of patients receiving CAR-T-cell therapy. The majority of CRS events were grade ≤2, occurring in 65% (32, 88) of TCE-treated patients and 39% (22, 60) of CAR-T-treated patients. Grade ≥3 CRS occurred in 3% (2, 7) of the TCE group versus 14% (2, 43) of the CAR-T group (*P* = 0.05).

**Table 3:** Treatment-related adverse events.

Variable, mean	T-cell engager therapy				CAR-T therapy				Overall				Subgroup
	Number of studies	Number of patients, (*N* or *n*/*N*)	Pooled value (95% confidence interval [CI])	*I* ^2^ (%)	Number of studies	No. of patients (*N* or *n*/*N*)	Pooled value (95% CI)	*I* ^2^ (%)	Number of studies	Number of patients (*N* or *n*/*N*)	Pooled value (95% CI)	*I* ^2^ (%)	*P*-value
**CRS (%)**	10	393/749	59 [25, 86]	95^a^	3	16/37	43 [28, 59]	0	13	409/786	55 [28, 79]	93	.44
**Grade ≤2 (%)**	6	230/416	65 [32, 88]	93^a^	2	9/23	39 [22, 60]	0	8	239/439	58 [34, 79]	90	.20
**Grade ≥3 (%)**	4	9/259	3 [2, 7]	0	1	2/14	14 [2, 43]		5	11/273	4 [2, 7]	0	.05^a^
**Neurological events^b^**	6	61/531	9 [4, 17]	82^a^	2	9/23	39 [22, 60]	41	8	70/554	12 [6, 23]	82^a^	<.0001^a^
**Grade ≤2 (%)**	3	15/311	5 [2, 14]	76^a^	1	1/9	11 [0, 48]		4	16/320	5 [2, 13]	64	.46
**Grade ≥3 (%)**	3	3/127	2 [1, 7]	0	1	1/9	11 [0, 48]		4	4/136	3 [1,8]	0	.18
**Hematological events^c^**	8	121/634	23 [7, 56]	80^a^	3	15/37	34 [8, 75]	82^a^	11	136/671	26 [10, 52]	81	.65
**Grade ≤2 (%)**	4	39/341	13 [1, 73]	94^a^	1	2/14	14 [2, 43]		5	41/355	13 [2, 60]	92	.96
**Grade ≥3 (%)**	5	50/381	15 [4, 43]	94^a^	3	9/37	2 [0, 93]		8	59/418	12 [3, 38]	91	.51
**Hepatic events^d^**	3	66/258	33 [12, 64]	93^a^	2	7/28	25 [12, 44]	38	5	73/286	29 [14, 44]	90	.63
**Grade ≤2 (%)**	2	28/125	31 [4, 84]	96^a^	2	4/28	14 [5, 32]	0	4	32/153	22 [8, 50]	89	.47
**Grade ≥3 (%)**	2	6/125	5 [2, 10]	59	1	3/14	21 [5, 51]		2	9/139	8 [3, 20]	68	.03^a^
**MSK and derm events^e^**	6	196/538	33 [24, 42]	82^a^	1	4/14	29 [8, 58]		7	200/552	32 [25, 41]	79	.76
**Grade ≤2 (%)**	2	26/111	23 [16, 32]	0	1	3/14	21 [5, 51]		3	29/125	23 [17, 31]	0	.87
**Grade ≥3 (%)**	1	12/97	12 [7, 21]	–	1	1/14	7 [0, 34]		2	13/111	12 [7, 19]	0	.57
**Renal events^f^**	5	67/345	15 [8, 25]	72^a^	1	3/14	21 [5, 51]		6	70/359	16 [10, 24]	65	.53
**Grade ≤2 (%)**	2	19/125	15 [10, 23]	41	1	2/14	14 [2, 43]		3	21/139	15 [10, 22]	0	.93
**Grade ≥3 (%)**	1	5/137	4 [2, 8]	52					1	5/137	4 [2, 8]		NA
**GI events^g^**	7	132/554	21 [12, 36]	90.6^a^	1	2/14	14 [2, 43]		8	134/568	20 [12, 33]	75	.57
**Grade ≤2 (%)**	3	43/165	23 [11, 44]	79^a^	1	2/14	14 [2, 43]		4	45/179	21 [11, 38]	72	.51
**Grade ≥3 (%)**	2	3/75	4 [1, 12]	0					2	3/75	4 [1, 12]		NA
**Fevers/infections**	4	122/317	46 [23, 71]	92^a^	1	5/14	36 [13, 65]		5	127/331	44 [24, 65]	89	.59
**Grade ≤2 (%)**	1	31/97	32 [23, 42]						1	31/97	32 [23, 42]		NA
**Grade ≥3 (%)**													
**Fatigue**	7	221/578	38 [30, 45]	73^a^	2	10/28	36 [20, 55]	0	9	231/606	37 [31, 44]	65	.85
**Grade ≤2 (%)**	3	90/311	29 [24, 36]	44	2	7/28	25 [12, 44]	38	5	97/339	29 [23, 35]	24	.65
**Grade ≥3 (%)**	4	19/325	6 [3, 14]	69^a^	2	3/28	11 [3, 28]	0	6	22/353	7 [3, 13]	52	.43

a Significant data heterogeneity present (*P* ≤ .05).

b Includes ICANS, syncope, mental status change, dizziness, headaches, blurry vision.

c Includes leukopenia, prolonged aPTT, macrophage activation syndrome, thrombocytopenia, lymphopenia, neutropenia, anemia.

d Includes hypoalbuminemia, transaminitis.

e Includes fatigue, maculopapular rash, generalized nonspecific rash, arthralgia, back pain, extremity pain, myalgias, pruritis.

f Includes hypocalcemia, hypophosphatemia, hyponatremia, hypokalemia, bladder spasms, noninfectious cystitis, hematuria, proteinuria.

g Includes abdominal pain, anorexia, nausea, vomiting, diarrhea, constipation, dyspepsia.

#### Neurotoxicity and ICANS

Neurological TRAEs were reported in 9% (4, 17) of TCE patients and 39% (22, 60) of CAR-T patients (*P* < 0.0001). However, there was no significant difference between the two groups when comparing rates of grade ≤2 and grade ≥3 neurologic events. Immune effector cell-associated neurotoxicity syndrome was specifically reported in 3 trials: 1 trial reported a grade 5 event and a grade 2 events, 1 trial reported a grade 4 event and 2 grade 2 events, and 1 trial reported 2 grade 1-2 events. There was insufficient granularity in the extracted neurotoxicity data to pool the ICANS data separately.

#### Other toxicities

Hematological TRAEs were reported in 23% (95% CI: 7, 56) of patients receiving TCEs and 34% (8, 75) of those receiving CAR-T therapy. Hepatic events were observed in 33% (12, 64) of patients treated with TCEs and in 25% (12, 44) of CAR-T-treated patients. There was no significant difference between the groups in the incidence of grade ≤2 hepatic events; however, grade ≥3 hepatic events were more frequent in the CAR-T cohort (21%; 5, 51) compared to the TCEs group (5%, 95% CI: 2,10; *P* = 0.03) There was one grade 5 hepatitis event reported with a PSMA CAR-T. Renal TRAEs were reported in 15% (8, 25) of TCEs patients and 21% (5, 51) of CAR-T patients, with no significant differences observed between the two groups for any grade renal events. Musculoskeletal and dermatological (MSK/D) TRAEs were observed in 33% (24, 42) of patients receiving TCEs and 29% (8, 58) of those receiving CAR-T therapy, with no significant difference between cohorts at any grade. Gastrointestinal (GI) TRAEs were reported in 21% (12, 36) of TCEs patients and 14% (2, 43) of CAR-T patients. Fever-and infection-related TRAEs were documented in 46% (23, 71) of TCEs patients and 36% (13, 65) of CAR-T patients; no grade ≥3 events were reported. Treatment-related fatigue occurred in 38% (30, 45) of patients undergoing TCEs and 36% (20, 55) of patients receiving CAR-T therapy. Grade ≥3 fatigue was observed in 6% (3, 14) of TCEs patients and 11% (3, 28) of CAR-T patients. No significant difference in grade ≤2 fatigue was identified between the two groups.

### Clinical outcomes

Table 4 presents the pooled clinical outcomes of patients treated with TCEs and CAR-T therapy. Data on treatment discontinuation for any reason were available only in the TCE cohort, with a pooled discontinuation rate of 23% (CI: 3, 69) across 5 studies. Discontinuation due to AEs occurred in 7% (3, 19) of TCE-treated patients. Dose reductions or interruptions due to AEs were reported in 22% (7, 48) of TCE-treated patients and 11% (4, 29) in those receiving CAR-T therapy, though this difference was not statistically significant. Pooled mortality rates did not differ significantly between the two groups, with 5% (0%-56%) in the TCEs cohort and 11% (0%-48%) in the CAR-T cohort (*P* = 0.68), though interpretation is limited by substantial heterogeneity and small sample sizes.

**Table 4:** Outcomes.

	T-cell engager therapy				CAR-T therapy				Overall				Subgroup
Variable, mean	No. of studies	No. of patients	Pooled value (95% CI)	*I* ^2^ (%)	No. of studies	No. of patients (*N* or *n*/*N*)	Pooled value (95% CI)	*I* ^2^ (%)	No. of studies	No. of patients (*N* or *n*/*N*)	Pooled value (95% CI)	*I* ^2^ (%)	*P*-value
		(*N* or *n*/*N*)											
**Dose discontinuation for any reason (%)^b^**	4	68/357	23 [2, 81]	72^a^					4	68/357	23 [2, 81]	72^a^	NA
**Dose discontinuation due to adverse events (%)**	4	30/357	7 [2, 22]	77^a^					4	30/357	7 [2, 22]	77^a^	NA
**Dose reduction or interruption due to adverse events (%)**	5	89/475	22 [7, 52]	93^a^	2	3/27	11 [4, 29]	0	8	92/402	16 [6, 38]	89	.39
**Death**	3	9/96	5 [0, 56]	79^a^	1	1/9	11 [0, 48]	–	4	10/105	7 [1, 39]	72	.68

a Significant data heterogeneity present (*P* ≤ .05).

b Additional reasons include adverse events, consent withdrawal, and progressive disease.

## Discussion

Immuno-oncology has revolutionized the treatment paradigm of various malignancies, but has yet to have a transformative impact on patients with PC. Adoptive T-cell strategies have historically been hindered by their toxicity profiles, particularly in solid tumor malignancies.^37^ T-cell redirecting therapy, in the form of TCEs and CAR-T therapy, is at the initial stages of clinical development for patients with metastatic PC, and early data hold promise for these modalities, with recent late-phase trials now in progress.^38^ Novel TCEs and CAR-T constructs integrate mechanistic advantages to reduce off-target toxicity and improve efficacy. However, there has not been a thorough evaluation of the current literature to determine the toxicity profile of these therapies in this patient population. In this first multi-study systematic review and meta-analysis, we report on the safety profiles of TCEs and CAR-T in mCRPC patients.

Both therapeutic classes involve T-cell activation, and their adverse reactions are similar and related to T-cell hyperactivity, most notably CRS and ICANS. Cytokine release syndrome is a systemic inflammatory response that is mediated by cytokines, resulting in symptoms ranging from fever, malaise, and hypotension to multi-organ dysfunction and disseminated intravascular coagulopathy.^39^ Immune effector-cell-associated neurotoxicity syndrome occurs by a similar pathophysiologic mechanism but only involves neurologic symptoms, ranging from tremors and mild aphasia to encephalopathy, cerebral edema, and seizures.^40^

Our study noted that the incidence of all-grade CRS was comparable between the two groups, but the incidence of higher-grade CRS (grade ≥3) was higher in patients treated with CAR-T therapy. Furthermore, we noted higher rates of all grades of neurological TRAE (including ICANS) in CAR-T patients, although grade ≥3 rates were comparable. Hematological TRAEs were similar between the two cohorts, but grade **≥**3 events were higher in TCEs, although the difference did not reach statistical significance. Chimeric Antigen Receptor T-cell had significantly higher rates of hepatic events, such as transaminitis, than TCEs. Grade 5 toxicity events occurred with both classes of therapy, and were limited to two agents in particular. Two patients treated with a PSMA×CD28 TCE in combination with an anti-PD-1 antibody cemiplimab suffered from grade 5 immune-mediated AEs; hemophagocytic lymphohistiocytosis, and hepatitis.^35^ In a study evaluating a CART-PSMA-TGFβRDN construct, 2 patients suffered from grade 5 AEs; 1 immune-effector cell-associated neurotoxicity syndrome with associated macrophage activation syndrome, and 1 nonspecific immune-related toxicity. Correlative analyses from peripheral blood from both patients with grade 5 toxicities showed an elevated inflammatory signature compared to patients on study without significant toxicity, with higher levels of IL2, IL6, granulocyte-macrophage colony-stimulating factor, and IL-18.^17^^,^^22^

Most of our current insights into T-cell-engaging therapies’ efficacy and toxicity profiles have been derived from clinical trials in hematological malignancies. Both CAR-T therapies and TCEs have been evaluated in relapsed-refractory multiple myeloma (RRMM) and relapsed acute myelogenous leukemia (R-AML). Teclistamab, a TCE targeting B-cell maturation antigen (BCMA) and CD3, approved for RRMM, demonstrated all-grade CRS in 72.1% of patients, with only 0.6% experiencing a grade 3 AE. Similarly, all grade hematological TRAEs occurred in a 40%-70.9% range depending on cell lineage, with 21.2—64.2% experiencing a grade 3 or above event. Neurological TRAEs were observed in 14.5% of patients, of whom 3% experienced grade 1 or 2 ICANS, with no events exceeding grade 2 severity.^41^ Similarly, elranatamab, another BCMA x CD3 TCE, demonstrated grade 3/4 anemia and neutropenia rates of 37.4% and 48.8%, respectively, with CRS of any grade occurring in 57.5% of patients.^42^

T-cell-engaging therapies are still in early stages of development for patients with solid tumors. Substantial toxicity has been observed, as reflected in our study collection, reviewing patients with mCRPC. Collectively, findings from our analysis of mCRPC trials appear to parallel toxicity profiles seen in other solid tumor studies. This similarity may reflect the shared characteristics of TMEs in solid tumors, which have historically posed barriers to T-cell trafficking and activation but may also increase the risk of OTOT, in contrast to hematologic malignancies, where immune effector cell access to target cells is less restricted.

Our findings indicate that, while the incidence of all-grade CRS in patients with mCRPC receiving T-cell redirecting therapies is comparable to that observed in hematologic malignancies and other solid tumors, the rate of grade ≥3 CRS was notably lower in mCRPC. Similarly, patients with mCRPC also had lower rates of neurological TRAEs compared to those receiving approved therapies for RRMM and R-AML. Among patients treated with TCEs, hematological TRAEs—both all-grade and grade ≥3—were observed at comparable rates across mCRPC and hematologic malignancies. However, early studies of patients with mCRPC receiving CAR-T therapy experienced lower frequencies of hematological toxicities relative to patients with hematologic malignancies, while neurological TRAEs remained similar. These observations support the continued investigation of T-cell redirecting therapies in mCRPC, suggesting an acceptable toxicity profile. Although certain agents have been discontinued due to AEs, these findings indicate that, as therapeutic classes, both CAR-T and TCEs can replicate the clinical success achieved in hematologic cancers, provided that efficacy signals prove favorable.

Future research should aim to directly compare CAR-T and TCEs therapies targeting the same tumor antigen and to perform pooled toxicity stratified by target antigen. Given the differential expression patterns of tumor-associated antigens across healthy tissues, toxicity profiles are likely to vary significantly depending on the molecular target. On-target, off-tumor toxicities remain an inherent risk of T-cell-engaging strategies and are often uniquely associated with the targeted antigen. Most mCRPC-focused clinical trials have employed PSMA as the therapeutic target. Prostate-specific membrane antigen is a cell-surface glycoprotein overexpressed in prostate adenocarcinoma and maintained throughout disease progression. However, it is also expressed at lower levels in non-malignant tissues, including the salivary glands, renal tubular epithelium, small intestine, central nervous system, and neo-vasculature.^43^ Consequently, T-cell therapies targeting PSMA may carry an increased risk of neurotoxicity, GI toxicity, and CRS due to neo-vasculature and endogenous expression patterns, in contrast to agents targeting antigens with more restricted expression profiles.

If certain tumor antigens are found to be more immunogenic or associated with higher toxicity due to their expression profile, future therapeutic designs may benefit from engineering strategies aimed at reducing the affinity of the targeting domain—either to the tumor antigen or, in the case of TCEs, to the CD3ε subunit of the T-cell receptor. Moreover, while corticosteroids and tocilizumab currently represent the mainstays of CRS management, emerging strategies involving Janus kinase inhibitors, mTOR inhibitors, and neutralizing agents targeting TNFα and IFNγ may provide additional tools to mitigate high-grade CRS and other manifestations of T-cell hyperactivation.^44^

A key limitation of this meta-analysis lies in the small sample sizes of the individual trials that are included. Although a total of 861 patients across 13 phase I/II TCEs clinical trials and 55 patients across 5 CAR-T trials were included, most of these were early-phase, first-in-human studies, often reported only in abstract form without accompanying publications. This constrained the depth and granularity of the data available for extraction and modeling. In many cases, safety outcomes were reported in aggregate across multiple dose levels, precluding a dose-dependent toxicity analysis. These limitations reflect the early state of T-cell-engaging therapy development in solid tumors—a field that is now rapidly evolving. CAR-T therapy protocols often include lymphodepletion (LD) prior to CAR administration, and the specific LD regimen can influence the toxicities experienced by patients throughout treatment that are not directly attributable to the CAR itself. This study pooled reported toxicities from patients who received different LD regimens, dosing schedules, or no LD at all, making it impossible to distinguish the contribution of each component of care to the observed toxicities based on the published data. Moreover, the analysis includes outcomes from patients treated with both TCEs and CAR-T therapies incorporating different co-stimulatory domains, as well as TCEs with distinct single-chain variable fragments (e.g., CD3+, CD28+) that vary in effector cell affinity—factors that can influence both the type and severity of toxicities observed. Lastly, while this study pooled efficacy outcomes from T-cell redirecting therapies reported to date, these results should be interpreted with caution due to heterogeneity and limited sample sizes. The included trials represent a variety of mechanisms of action, molecular targets, and patient populations, thereby limiting the ability to draw meaningful conclusions about comparative efficacy. Therefore, this study serves as a pooled safety analysis of the initial data from the initial experience of these classes of therapy in PC, and provides a basis for further investigations as trials progress and more granular data are collected.

In summary, in patients with mCRPC, TCEs and CAR-T therapies demonstrate comparable rates of hematological, musculoskeletal/dermatologic, renal, and GI TRAE. However, CAR-T therapy is associated with significantly higher incidences of all-grade neurologic toxicity, as well as grade ≥3 CRS and hepatic TRAEs. As clinical investigations into TCEs and CAR-T therapy in mCRPC continue to expand, the breadth of data generated will yield more robust, granular datasets, facilitating a more comprehensive understanding of safety and efficacy. This meta-analysis consolidates the existing literature and provides a foundational reference for characterizing AEs profiles associated with T-cell-redirecting therapies. These findings can inform the design of future clinical trials, enabling researchers and clinicians to anticipate and manage class-specific toxicities more effectively. Importantly, the data underscore the need to recognize the distinct safety profiles of TCEs and CAR-T therapies, and to maintain appropriate clinical vigilance throughout treatment.
